# Perception of the ethical climate among hospital employees in a public healthcare system: a cross-sectional survey at the University Hospital of Split, Croatia

**DOI:** 10.1186/s12910-025-01217-1

**Published:** 2025-05-07

**Authors:** Zrinka Hrgović, Luka Ursić, Jure Krstulović, Marin Viđak, Ljubo Znaor, Ana Marušić

**Affiliations:** 1 Department of Family Medicine, Health Center of Split– Dalmatia County, Split, Croatia; 2 University of Split School of Medicine, Split, Croatia; 3 Department of Research in Biomedicine and Health, Centre for Evidence-based Medicine, University of Split School of Medicine, Split, Croatia; 4 Department of Health Care Quality, University Hospital of Split, Split, Croatia; 5 Department of Surgery, University Hospital of Split, Split, Croatia; 6 Department of Cardiology, Dubrava University Hospital, Zagreb, Croatia; 7 Department of Ophthalmology, University Hospital of Split, Split, Croatia; 8 Department of Ophthalmology, University of Split School of Medicine, Split, Croatia; 9 Department of Research, University Hospital of Split, Split, Croatia

**Keywords:** Ethics, Ethical climate, Hospital, Ethical climate questionnaire

## Abstract

**Background:**

In this cross-sectional study, we assessed the ethical climate at the University Hospital of Split in Croatia and investigated its potential indicators.

**Methods:**

We used a validated Croatian translation of the 36-item Ethical Climate Questionnaire, which we distributed online (via an e-mail sent by the hospital administration to hospital employees) and as a paper and pen survey directly to all hospital departments. We compared ECQ scores between doctors of medicine (MDs)/doctors of dental medicine (DMDs) and other employees; MDs/DMDs and nurses; employees working with patients and those not working with patients; and employees working in the ICU versus those not working in the ICU using the Mann-Whitney U test. We used linear regression to explore the relationship of each ethical climate with gender, age, degree level, and years spent working in the hospital.

**Results:**

We collected 325 physical and 222 online questionnaires (547 responses in total), after which we excluded 146 incomplete responses. This left 401 questionnaires for analysis, primarily from doctors (*n* = 175; 43.6%) and nursing staff (*n* = 131; 32.7%). The two dominant climates were ‘Company rules’ and ‘Laws and professional codes’. Stratified by profession, we observed higher scores for ‘Personal morality’ among doctors of medicine or dental medicine, whereas the group comprising other health professionals and non-medical staff had higher scores for ‘Team interests’, ‘Efficiency’, ‘Social responsibility’, and ‘Laws and professional codes’. In comparing nurses and doctors of medicine/dental medicine, we observed the former group had higher scores for ‘Social responsibility’, ‘Efficiency’, and ‘Team interest’, while the latter had higher scores for ‘Personal morality’. Those who worked outside of the ICU had higher scores for ‘Social responsibility’ compared to those who did not. In the regression analyses, age was a significant positive predictor of the ‘Laws and professional codes’ climates, and years spent working in the hospital acted as a positive predictor of the ‘Self-interest’ climate.

**Conclusion:**

A large university hospital center in a fully publicly funded national healthcare system has a positive ethical work climate, which could be further developed by further development and implementation of codes of ethics to outline expected behaviors from all employees.

**Supplementary Information:**

The online version contains supplementary material available at 10.1186/s12910-025-01217-1.

## Introduction

The ethical climate is a way of understanding the influence of organisational practices and procedures on employees’ ethical beliefs and behaviours [1]. It represents the general characteristic of an organisation, thus affecting a broad spectrum of decisions [2], and encompasses the perception of what ‘right’ behaviour entails, making it a psychological tool for facing and resolving ethical issues [3]. It is also a type of organisational climate, encompassing employees’ perceptions of policies, practices, and procedures, as well as behaviours that they perceive as being expected and rewarded in their context [4]. Research has demonstrated that the intra-organisational environment is a major factor in ethical decision-making [5, 6]; therefore, if an organisation employs ethical decision-making, its employees will probably behave in line with pre-defined rules of ethical conduct [7]. Studies have also suggested that individuals’ perception of ethical work climate largely affects their behavioural intentions [5, 8, 9]. Furthermore, ethical climate dimensions have been shown to correlate separately with ethical behaviour, demonstrating how undesirable behaviours within workgroups could be corrected [10, 11].

**Fig. 1:**
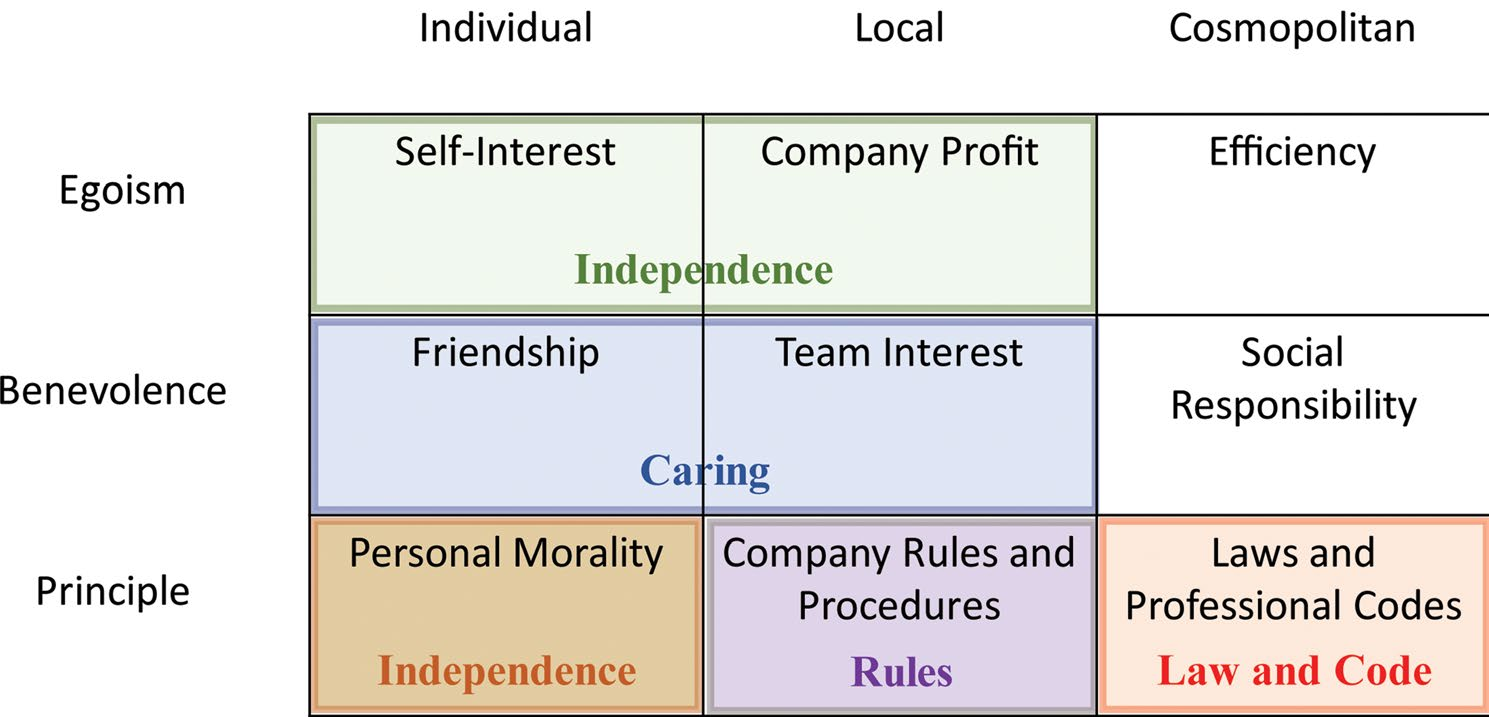
Theoretical ethical climates according to the ethical theory and the locus of analysis (in black font), and five common empirical derivatives of ethical climate (in coloured font), according to Martin & Cullen, 2006.

Victor and Cullen designed a two-dimensional theoretical typology of ethical climates, based on Kohlberg’s theory of moral development [2]. They developed and later revised the Ethical Climate Questionnaire (ECQ), a popular instrument for examining the ethical climate within an organisation [12]. The first, ‘ethical criterion’ dimension of this questionnaire is based on moral reasoning and includes egoism (maximising self-interest), benevolence (maximising community interests), and deontology (compliance with the principles). The second dimension, called the ‘locus of analysis’, identifies the sources of ethical reasoning within the organisation and comprises three categories: individual, local, and cosmopolitan. Consequently, cross-classification of the ethical criterion and locus of analysis criteria generates nine theoretical climate types (‘Self-interest’, ‘Company profit’, ‘Efficiency’, ‘Friendship’, ‘Team interest’, ‘Social responsibility’, ‘Personal morality’, ‘Company rules’, and ‘Laws and professional codes’), five of which were empirically derived and identified in practice [3, 13] (Fig. 1). In an instrumental climate, behaviour is guided by one’s own or the company’s interest. A caring climate is based on concern for others, which is embedded in the policies and practices of the organisation. An independence-based climate indicates that individuals follow their own personal and moral beliefs despite external mediators. A law and codes-based climate is one in which an organisation supports principled decision-making based on external legal and professional codes of conduct. A rules-oriented climate is expected to be guided strictly by company rules and procedures [2, 3].

The organisational ethical climate has been implemented in business [3, 14, 15] and recently in academic institutions [16, 17]. However, there has been growing interest in ethical climate within healthcare contexts, especially in nursing literature. Victor and Cullen’s typology of ethical climates and ECQ were used in some studies on the most common types of ethical climates in nursing workplaces. While some of them evaluated the correlation of each of the prevailing climate types with employment characteristics of nurses [18], others examined the relationship between the prevailing ethical climate and missed nursing care [19]; organisational culture, organisational commitment, job satisfaction, organisational citizenship behaviour, and turnover intentions [20–25]; moral distress [26]; ethical leadership and moral courage in crisis [27]; organisational trust and whistleblowing [28]; and teamwork [29]. Teymoori et al. [30] focussed on organisational culture and commitment among surgical team members (surgeons, anaesthesiologists, operating room nurses, and anaesthesia nurses). Another study [31] assessed the relationship of the ethical climate with employee performance and paternalistic leadership among all healthcare workers– physicians, nurses and administrative staff. This leaves a gap in knowledge about how all service providers in a hospital perceive the ethical climate, which would give insight into the difference between specific healthcare provider subgroups. Some studies specifically investigated the role of the intensive care unit (ICU) in the perception of the ethical climate. In a survey based on the self-assessment Ethical Decision-making Climate Questionnaire, Van den Bulcke [32] highlighted the significance of the ethical climate in ICUs for critical care physicians’ desire to leave their workplace. Furthermore, doctors in ICUs have been reported to have a more positive view of the ethical decision-making climate than nurses [33, 34]. According to Silverman et al. [34], nurses showed significantly higher levels of moral distress and intentions to leave their jobs than physicians. Also, other studies using the Hospital Ethical Climate Survey [1] found that a poor ethical climate, unintegrated end-of-life care teams, and a lack of nurse empowerment are related to increased moral distress in ICU workers [35, 36]. Meanwhile, research has shown that nurses working in the ICU perceived their ethical climate more positively than those in other units, possibly due to more intense interactions among healthcare professionals who face ethically sensitive issues [37, 38].

To address the gap in research regarding different health professionals and work settings, we assessed the ethical climate at the University Hospital of Split in Croatia by using a validated Croatian translation of the ECQ [16, 17]. We sought to explore the ethical climate among all of the healthcare staff at the hospital, while also examining the climate within the ICU specifically compared to the non-ICU context, as existing studies have done so using questionnaires other than the ECQ. We also investigated sociodemographic predictors, such as age, gender, highest educational qualification and years of employment, for the ethical climate.

## Methods

### Study design

This cross-sectional study was based on a survey of the perceptions of the ethical climate at the University Hospital of Split. We reported our findings according to the Consensus-based Checklist for Reporting of Survey Studies [39] (see Additional file 1).

### Setting

Croatia, the country in which the study hospital is based, has a mandatory social health insurance system with nearly universal population coverage [39], but still faces healthcare challenges related to the 1991–1995 Homeland War in Croatia, post-communist transition and contemporary problems of high mortality, low-quality monitoring, and the COVID-19 pandemic [40–43].

The University Hospital of Split is a public secondary/tertiary-level hospital which provides primary and specialised medical advice, conducts medical research, and provides clinical teaching at the University of Split School of Medicine. With 3,655 employees in 2024, divided among 15 departments, 9 divisions, 7 sections and sub-sections, as well as 1,500 acute and 30 chronic contractual beds and 24 surgical rooms, it is the largest hospital centre in the Split Dalmatia County, the second largest in Croatia. Twelve offices and the directorate perform routine and administrative work. Along with approximately half a million foreign visitors in the summer, the hospital serves about a million people from the Republic of Croatia and about half a million citizens of neighbouring Bosnia and Herzegovina [44].

### ECQ survey

The survey had two parts. The first collected basic demographic data, i.e. the respondents’ gender, age, highest educational qualification, additional scientific qualification, employment positions, and whether they worked in the ICU. The second consisted of the ECQ questionnaire [12, 16, 17], which has 36 items graded on a 6-point Likert-type response scale ranging from ‘0’ (completely false) to ‘5’ (completely true) (see **Additional file 2**). The score for each climate is obtained by summing the response to four specific items from the ECQ, as follows: ‘Self-interest’ = items 1, 6, 10, and 33; ‘Friendship’ = items 5, 16, 32, and 35; ‘Personal morality’ = items 3, 9, 11 and 22; ‘Company profit’ = items 4, 8, 17 and 29; ‘Team interest’ = items 12, 21, 27, and 31; ‘Company rules’ = items 7, 15, 18, and 23; ‘Efficiency’ = items 2, 19, 25, and 36; ‘Social responsibility’ = items 26, 28, 30, and 34; Laws and professional codes = items 13, 14, 20, and 24. The total score for each climate could range from ‘0’ to ’20’. The prevailing ethical climate was considered dominant when it received the highest score. The English version of the questionnaire has been back-translated to Croatian and validated in previous studies [12, 16, 17]. The overall Cronbach’s *α* in our sample was 0.897, comparable to that calculated in a previous study (α = 0.891) with the employees of the University of Split [16].

### Sample characteristics and inclusion/exclusion criteria

We used simple convenience sampling of the employees at the University Hospital in Split present at their workplace when the survey questionnaire was being distributed (October to December 2022), making all 3,655 individuals employed at the time of the study eligible for inclusion, irrespective of their gender, age, years of experience, or position within the hospital. Based on a confidence level of 95% and margin level of 5%, we aimed to include at least 348 participants, including doctors, nurses and other health professionals, as well as staff working within the administration, hospital infection service, medical physics department, scientific work department, cleaning and sterilisation service, nutrition and dietetics service, occupational safety department, IT service, and technical service department. The sample size was calculated using an online sample size calculator (https://www.calculator.net/sample-size-calculator.html).

### Survey administration

We first distributed the questionnaire *via* the hospital administration, which sent an e-mail containing the link to the online survey to all hospital employees. We did not send a follow-up reminder. We then distributed the physical versions of the questionnaire to all hospital departments and collected them after a few days in closed collection boxes to ensure anonymity. We also asked the employees not to fill in the paper questionnaire if they had responded to its online version, to avoid duplicate responses.

### Ethical considerations

The University Hospital of Split Ethics Committee (Document Class: 500-03/22 − 01/81, Reg. No. 2181 − 147/01/06/M.S.-22-02) and the University of Split School of Medicine (Document Class: 003–08/22 − 03/0003, Reg. No. 2181-198-03-04-22-0047) gave ethical approval for the study. We did not collect any personal data from the respondents. Both the online and physical questionnaires informed individuals on the full anonymity of the survey, with the former being programmed not to collect the IP addresses. The survey began with a notice informing individuals that, by continuing to answer the survey, they consent to participate.

### Statistical analysis

We described categorical variables with frequencies and percentages and continuous/ordinal ones with medians and interquartile ranges. We presented the results of the ECQ score with medians and interquartile ranges. We compared ECQ scores between doctors of medicine (MDs)/doctors dental medicine (DMDs) and other employees; MDs/DMDs and nurses; employees working in the ICU versus those not working in the ICU; employees working with patients and those not working with patients; medical and non-medical staff using the Mann-Whitney U test. We used enter linear regression (i.e. we inputted all dependent variables simultaneously) to examine the relationship of each ethical climate with gender, age, degree level, and years spent working in the hospital. *P*-values < 0.05 were considered as statistically significant. We performed all analyses in jamovi, version 2.3.6. (jamovi project, Sydney, Australia).

## Results

### Sample

We collected 325 physical and 222 online questionnaires (547 responses in total) and excluded 146 incomplete responses (of which 111 were online questionnaires), leaving 401 questionnaires for analysis. Considering that the hospital had 3,655 employees at the time of the study and that the e-mail survey likely reached them all, the response rate was 14.9%, with a completion rate of 73.3%. Most participants (*n* = 303; 75.6%) were female, with an overall median age of 39 years. Two-thirds of the respondents (*n* = 267, 66.6%) had a bachelor’s, master’s, or other university-level degree, and 178 (44.4%) were employed at the hospital for more than 10 years. The respondents were primarily MDs/DMDs (*n* = 175; 43.6%) and nursing staff (*n* = 131; 32.7%). Eighty-six (21.4%) worked in the ICU (Table 1).

**Table 1:** Participants’ demographic characteristics

Gender	*n* (%)
Male	98 (24.4)
Female	303 (75.6)
**Age in years**,** median (interquartile range)**	39 (31–50)
**Highest educational qualification**	
High school or lower	76 (19.0)
Bachelor’s, master’s, or another university-level degree (including MD/DMD)	267 (66.6)
PhD or equivalent*	58 (14.4)
**Years of employment**	
< 1 year	37 (9.2)
1–4	100 (24.9)
5–10	86 (21.4)
> 10 years	178 (44.4)
**Type of working staff**	
MD/DMD*	175 (43.6)
Nursing staff	131 (32.7)
Laboratory staff	11 (2.7)
Physical therapy staff	17 (4.2)
Non-medical staff	67 (16.7)
**ICU staff**	
Yes	86 (21.4)
No	315 (78.6)

DMD– doctor of dental medicine, ICU– intensive care unit, MD– medical doctor

*Includes one DMD

### Ethical climate scores

The two dominant climates were ‘Company rules’ and ‘Laws and professional codes’, meaning they had the same score without a statistically significant difference between them (Table 2).

**Table 2:** Perceived ethical climate at the University Hospital of Split

Ethical climate dimension (score range 0–s20)	Median score (interquartile range)
Self-interest	12 (9–14)
Friendship	12 (9–14)
Personal morality	11 (9–13)
Company profit	11 (9–13)
Team interest	11 (8–14)
Company rules*	13 (11–15)
Efficiency	12 (10–14)
Social responsibility	13 (10–15)
Laws and professional codes*	13 (11–15)

*Dominant ethical climates, i.e. those with the highest score and with no significant difference in the score between them. Mann-Whitney test

Stratified by profession, we observed higher scores on ‘Personal morality’ among the MDs/DMDs compared to other health professionals (nurses, laboratory and physiotherapy staff, and non-medical staff). However, the latter group had higher scores for ‘Team interest’, ‘Efficiency’, ‘Social responsibility’, and ‘Laws and professional codes’, although these differences were marginally significant. In comparing nurses and MDs/DMDs, we observed that the former group had higher scores for ‘Social responsibility’, ‘Efficiency’, and ‘Team interest’, while the latter had higher scores for ‘Personal morality’. Those who worked outside of the ICU had higher scores for ‘Social responsibility’ compared to those who did not. Finally, we found no differences in ECQ scores between those who worked with patients and those who did not, and a marginally significant difference in the ‘Friendship’ climate for individuals in the medical vs. non-medical profession, with the former having higher scores (Table 3).

**Table 3:** Comparison of ethical climate scores among different groups of professionals

Ethical climate dimension (score range 0–20)	ECQ score, median (interquartile range)		*P*-value†
MD/DMD vs. others*	MDs/DMDs (*n* = 175)	Others (*n* = 226)	
Self-interest	12 (8.5–14)	12 (9–13)	0.436
Friendship	12 (8–14)	12 (9–14)	0.278
Personal morality	12 (10–14)	11 (9–13)	0.007
Company profit	11 (9–13)	11 (9–13)	0.161
Team interest	11 (7–13)	12 (9–14)	0.017
Company rules	13 (10–15)	13 (11–15)	0.062
Efficiency	12 (9–14)	12 (11–14)	0.040
Social responsibility	12 (9.5–15)	13 (11–15)	0.023
Laws and professional codes	13 (10–15)	14 (11–15)	0.022
**MD/DMD** vs. **nurses**	**MDs/DMDs (** ***n*** ** = 175)**	**Nurses (** ***n*** ** = 131)**	
Self-interest	12 (8.5–14)	12 (9–13)	0.920
Friendship	12 (8–14)	12 (10–15)	0.064
Personal morality	12 (10–14)	11 (8–13)	0.022
Company profit	11 (9–13)	11 (10–13)	0.060
Team interest	11 (7–13)	12 (9–14)	0.013
Company rules	13 (10–15)	13 (11.5–15)	0.147
Efficiency	12 (9–14)	13 (11–15)	0.004
Social responsibility	12 (9.5–15)	13 (11–15)	0.020
Laws and professional codes	13 (10–15)	14 (11.5–15)	0.055
**Working in ICU**	**Yes (** ***n*** ** = 86)**	**No (** ***n*** ** = 315)**	
Self-interest	12 (9–14)	12 (9–14)	0.375
Friendship	11 (8–14)	12 (9–14)	0.394
Personal morality	11 (10–13)	11 (9–13)	0.995
Company profit	11 (8–12)	11 (9–13)	0.194
Team interest	11 (7–13)	12 (9–14)	0.114
Company rules	13 (10–14)	13 (11–15)	0.097
Efficiency	12 (9–14)	12 (10–14)	0.266
Social responsibility	12 (9–14)	13 (11–15)	0.019
Laws and professional codes	12.5 (10–15)	13 (11–15)	0.054
**Working with patients**	**Yes (** ***n*** ** = 312)**	**No (** ***n*** ** = 89)**	
Self-interest	12 (9–14)	12 (9–14)	0.323
Friendship	12 (9–14.3)	10 (9–13)	0.031
Personal morality	11 (10–13)	11 (9–13)	0.097
Company profit	11 (9–13)	12 (9–13)	0.251
Team interest	12 (9–14)	11 (7–13)	0.351
Company rules	13 (10–15)	14 (11–16)	0.082
Efficiency	12 (10–14)	12 (11–14)	0.884
Social responsibility	13 (10–15)	13 (10–15)	0.747
Laws and professional codes	13 (11–15)	14 (12–16)	0.089
**Medical vs. non-medical profession**	**Medical (** ***n*** ** = 334)**	**Non-medical (** ***n*** ** = 67)**	
Self-interest	12 (9–14)	12 (8.5–14)	0.795
Friendship	12 (9–14)	10 (9–12.5)	0.041
Personal morality	11 (9–13)	11 (9–13)	0.113
Company profit	11 (9–13)	11 (8–13)	0.768
Team interest	12 (8.25–14)	10 (7–14)	0.456
Company rules	13 (10.3–15)	13 (11–15)	0.777
Efficiency	13 (10–15)	12 (11–13)	0.121
Social responsibility	13 (10–15)	12 (10–14)	0.575
Laws and professional codes	13 (11–15)	14 (11–15)	0.724

DMD– doctor of dental medicine, ECQ– Ethical Climate Questionnaire, ICU– intensive care unit, MD– doctor of medicine

*Includes nursing, laboratory, physical therapy, and non-medical staff

†Mann-Whitney U test

We found that age was a significant negative predictor of the ‘Self-interest’ and a positive predictor of the ‘Laws and professional codes’ climates. Years spent working in hospital acted as a negative predictor for the ‘Team interest’ and ‘Laws and professional codes’ climates and as a positive predictor of the ‘Self-interest’ climate. While we observed other relationships between these two variables and the ‘Company profit’, ‘Company rules’, and ‘Social responsibility’ climates, they were marginally significant and accounted for a low level of variance within the data (R^2^ values of 0.0256, 0.0231, and 0.0376, respectively). Otherwise, we only found gender to be a significant negative predictor of the ‘Efficiency’ climate. However, these relationships should be interpreted with caution, as all regression models explained a low level of variance in the data (see Table S1 in the **Additional file 3**).

## Discussion

Our study aimed to explore the organisational ethical climate in a healthcare institution in a public healthcare system.

### Findings of the study

We found that the dominant ethical climates among all of the University Hospital of Split employees were ‘Company rules’ and ‘Laws and professional codes’. Also, MDs and DMDs scored higher on the ‘Personal morality’ ethical climate than the other non-medical and medical staff, as well as nurses specifically. However, they scored lower on the ‘Team interests’, ‘Efficiency’, ‘Social responsibility’, and ‘Laws and professional codes’ than all other staff, and on the ‘Social responsibility’, ‘Efficiency’, and ‘Team Interest’ climates than nurses specifically. Regarding ‘Social responsibility’, individuals who worked outside of the ICU scored higher than those who did not. We did not find any differences in ECQ scores between the individuals who worked with patients and those who did not.

### Relevance of the study

Our study is unique because it looked at the ethical climate of the whole organisation, as perceived by all hospital staff. The two dominant climates at a public hospital, ‘Company rules’ and ‘Laws and professional codes’, share a principle construct based on deontology, but differ in their local and cosmopolitan locus of analysis [13]. Both are fundamentally considered ‘principled’ climates, whereby organisational or group norms advise that in ethically challenging situations, the decision-maker adheres to rules and codes [12, 45]. Studies have shown that, in contexts where both of these principled climates exist, employees conduct themselves under established rules and legal regulations [11, 15, 46]. These climates have also been labelled as ‘normative’ [15], and have been considered as ‘positive’ due to their positive impact on employees’ performance and negative correlation with dysfunctional behaviour [3, 47]. In some studies, the ‘Laws and professional codes’ climate was referred to as the ‘professionalism’ climate [18, 22, 30]. Some authors [26] suggest that the ‘Company rules’ and ‘Laws and professional codes’ should be a single climate type– ‘Rules’, while Saygili [31] calls them ‘Principles’.

The predominant climates at the study hospital were identical to those observed at the University of Split School of Medicine [17], to which it is directly connected through daily educational and research activities. Both institutions are almost solely publicly funded. Likewise, both fall within the legal and financial purview of the Government, but under different branches: the Ministry of Science and Education funds the university and the Ministry of Health oversees the hospital. Additionally, most of our respondents were MDs who graduated from the University of Split and are its faculty for the clinical part of the medical curriculum. These factors possibly jointly contributed to the finding that the ‘Company rules and procedures’ and ‘Laws and professional codes’ were the dominant climates at both institutions.

Additionally, our finding that most demographic characteristics (aside from age and years spent working in hospital for the ‘Laws and professional codes’ climate) were not or were only marginally predictive of the dominant ethical climate suggests that it depends on factors that may be external to the institution. This suggests that, for future policies, guidance and training regarding workplace climate in a public hospital, more general measures coming outside of the organisation could be more effective than interventions targeting individual employees. However, our results on the differences between different professionals provide insight into how various groups of healthcare workers view and prioritise ethical principles and values in their work environment. For example, the higher scores for ‘Personal morality’ among MDs/DMDs than in other groups suggest a need for integrating ethical discussions and education tailored to their specific values and dilemmas. Higher scores for ‘Team interests’, ‘Efficiency’, ‘Social responsibility’, and ‘Laws and professional codes’ among other healthcare workers suggest that they may be more oriented towards collective aspects of care and institutional policies. Furthermore, differentiating between the ethical climates that employees encounter outside of ICUs and those within them offers important information on how particular obstacles and working settings affect ethical priorities and perceptions. Considering all of these differences is important in creating tailored and effective ethics training programmes, policies, and practices.

We included all hospital employees, as they are all involved in its daily functioning, allowing for a more robust insight into the organisational ethical climate. We confirmed that climates related to general principles are dominant in hospitals, as has been shown in studies involving only nurses, where ‘Company rules’ [21] or ‘Laws and professional codes’ [23, 48] were predominant. These results show how important it is for healthcare facilities to adhere to policies, procedures, and professional standards, possibly because healthcare work is essential and carries ethical responsibilities.

We can also compare our study to those conducted in other healthcare systems. For example, a study from Egypt [21] found that, while the ‘Rules’ climate was dominant both among nurses employed in a hospital affiliated with the private health sector and in public hospitals, it was perceived as significantly ‘more dominant’ in the private hospital context. This was explained by incentives (‘rules’) from the private hospital’s administration fostering a positive ethical environment to retain nurses and increase financial stability and profit. Meanwhile, a study from the Republic of Cyprus [19] found minor or no significant differences in the prevailing climates between public hospitals, non-profit non-governmental hospices, and independent oncology centres that provide nursing care for cancer patients. This suggests that certain strategies or policies may be universally effective in cultivating a positive ethical climate, regardless of the organisational context. Overall, comparing ethical climates across different healthcare systems helps identify best practices, inform policy development, and ultimately improve the quality of care.

Our findings are most comparable to those of Dinc and colleagues [23], involving nurses from Bosnia and Herzegovina, with which the Croatian healthcare system historically shared a communist-controlled market in former Yugoslavia. After the war in 1990s, the two countries adapted differently to the market economy, which in turn affected their healthcare systems. Croatia continued with the universal healthcare system funded through mandatory health insurance of the working population [40], while Bosnia and Herzegovina ended up with 13 different healthcare reimbursement systems and with more significant participation of the private sector as a consequence of the political division of the country [49]. Dinc et al. [23]. conducted their study in three different cantons, each with its own Ministry of Health in charge of the organisation of the healthcare system at primary, secondary, and tertiary levels, as well as private healthcare institutions competing with the public institutions. The study participants– nurses working in three public and one private institution– perceived the ‘Laws and professional codes’ climate to be the dominant one in their context. In contrast to our research, their investigation was limited only to nurses and was carried out in a decentralised healthcare system.

Regarding the relationship between employees’ sociodemographic characteristics and the perceived ethical climate, we found older age was a positive predictor of the ‘Laws and professional codes’ climate, while more years spent working in the hospital negatively predicted this climate. This is in contrast with the findings of Abou Hashis [21] that younger nurses (< 30 years old) had significantly higher mean score for the perception of a positive ethical climate. Meanwhile, Vryonides et al. [19] showed that nurses < 25 years of age had statistically significant lower mean values for the ‘Caring’ climate than those aged 35–54 years. It is difficult to make conclusions about the role of age and the duration of employment from these results, and this requires further studies which could inform interventions to increase positive ethical climate among different age groups.

Our finding that educational level played no role as a predictor for the ethical climates also diverges from previous research. Vryonides et al. [19] found that nurses with a bachelor’s degree had statistically significant higher mean score on ‘Rules’ climate than those with an MSc or higher (such as a PhD). They also found that males had statistically higher mean score for ‘Independence’ ethical climate than females. Moreover, we found gender only negatively predicted the ‘Efficiency’ climate. This is possibly because the nature of principled climates, characterised by adherence to rules and professional codes, makes them universally applicable and equally emphasised across genders within healthcare workers.

Our study may be a useful starting point for cross-system comparisons to explore how ethical climates differ in various healthcare systems, including those with private and hybrid models. Future research could also investigate the relationship between ethical climate and key healthcare outcomes such as patient satisfaction, employee retention, and clinical decision-making. The observed differences in ethical climate perceptions among different professional groups suggest the need for further study into how ethical training and professional socialisation shape ethical attitudes, which could inform the tailoring of ethics education programmes to different healthcare professionals. Finally, given the global relevance of ethical climate in healthcare, longitudinal studies could track changes over time, while cross-cultural research could identify universal and system-specific ethical climate determinants.

### Strengths and limitations

This study’s strengths include its comprehensive examination of the ethical climate in a healthcare setting, large sample, its inclusivity and coverage of diverse staff demographics, and the use of a validated questionnaire.

Our study also has some limitations. The ECQ is based on self-reporting and may be subject to social desirability and other biases inherent to self-reported questionnaires. We tried to mitigate this by fully anonymising the survey. As we distributed the survey in two formats (online and paper), it is possible that some participants answered twice; however, we believed that this is not likely for busy hospital employees, especially as they have been warned not to fill in the physical questionnaire if the completed the online one, and vice versa. Additionally, the ECQ has been validated and measured mostly in business settings and has no questions specific for healthcare institutions, although it has been applied in healthcare settings [50]. Likewise, its application at the University of Split School of Medicine [16, 17] allows for a direct comparison of our findings, as the two institutions share a cultural context, activities, and employee base. The psychometric characteristics of the Croatian ECQ used in this study was similar to that from the study with University of Split employees [16].

## Conclusions

Our study provides valuable insights into the ethical climate of a public healthcare institution operating within a universal health coverage system. Our findings may have broader implications for policymakers who are interested in improving organisational ethics, healthcare quality, and policy-making in public healthcare systems worldwide. The prevalence of rule-based ethical climates suggests that healthcare institutions rely heavily on regulation and professional codes. Policymakers should strengthen ethical governance by ensuring that ethical standards are not only inclusive, but also appropriately implemented and updated frequently. As the ethical climate affects employee behavior and job satisfaction, policymakers could incorporate ethical climate surveys into hospital accreditation criteria and performance reviews. This would allow continuous monitoring and enhancement of ethical practice within healthcare facilities. Because different professional groups have distinct ethical climate perceptions, training programmes need to be tailored to meet their own unique ethical issues. For example, medical physicians, nurses, and administrative staff may undergo special training that suits their unique responsibilities and ethical issues they face in their work. Our results also indicate that the organisational ethical climate is shaped by external factors outside of one’s demographic characteristics. This means that policy actions at the national and institutional levels aimed at shaping workplace ethics should be aimed at systemic intervention rather than at individual employees. Comparison of ethical climate studies across different healthcare systems may allow policymakers to acquire cross-institutional best practices from public and private institutions. For example, policymaking in countries considering integrating healthcare ethics and governance could be guided by lessons from systems with strong rule-based climates.

## Electronic supplementary material

Below is the link to the electronic supplementary material.

Supplementary Material 1: Additional file 1.docx Checklist for Reporting Of Survey Studies (CROSS)

Supplementary Material 2: Additional file 2.docx Ethical Climate Questionnaire

Supplementary Material 3: Additional file 3.docx Linear regression analysis of sociodemographic variables

## Data Availability

The data that support the findings of this study are openly available in the Open Science Framework at https://osf.io/y2meq/.

## Abbreviations

DMD: Doctor of dental medicine
ECQ: Ethical Climate Questionnaire
ICU: Intensive care unit
MD: Doctor of medicine
