# Supplementary material for: Perception of the ethical climate among hospital employees in a public healthcare system: a cross-sectional survey at the University Hospital of Split, Croatia
Source: BMC Med Ethics. 2025 May 7;26:59. doi: 10.1186/s12910-025-01217-1 (PMC12060318; doi:10.1186/s12910-025-01217-1)
Supplement: Supplementary file 2 — Supplementary Material 2: Additional file 2.docx Ethical Climate Questionnaire [file 12910_2025_1217_MOESM2_ESM.docx]

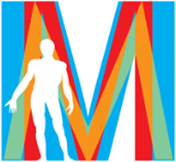
9996666

**University of Split**

**School of medicine**

**Universitas Studiorum**

**Spalatensis**

**Facultas Medica**


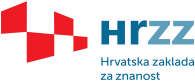


Dear Sir/Madam,

The purpose of this questionnaire is to determine changes in the assessment of the ethical climate among employees of the University Hospital of Split. This study builds upon similar research conducted at the School of Medicine and the Faculty of Philosophy at the University of Split (Viđak M et al, Sci Eng Ethics 2020). The research has obtained ethical approvals from the relevant institutions.

The questionnaire consists of two parts. In the first part, we kindly ask you to provide some information about yourself, followed by the questionnaire regarding your attitudes towards the ethical climate in your institution. All data from this questionnaire are collected completely anonymously and will be analyzed only at the group level, ensuring that your anonymity is never compromised. The electronic version of the questionnaire will not collect information about your IP address, thus ensuring complete anonymity.

By continuing to fill out the questionnaire, you consent to participate in the research.

**Gender** (select) M F **Age** (in years) ______

**Working place?**

|  | Clinic for Anesthesiology, Reanimatology and Intensive Care |  | Department of Clinical Epidemiology |
| --- | --- | --- | --- |
|  | Clinic for heart and cardiovascular diseases |  | Medical Physics Department |
|  | Department of Otorhinolaryngology |  | Department of Health Care Quality |
|  | Department of Pediatrics |  | Science Department |
|  | Pediatric Surgery Clinic |  | Department of Central Sterilization |
|  | Clinic for Infectious Diseases |  | Hospital cleaning Department |
|  | Surgery Clinic |  | Department for Finance and Economics |
|  | Clinic of Dermatovenereology |  | Department for Investments and Development (EU projects) |
|  | Department of Neurology |  | Public Procurement Department |
|  | Department of Ophthalmology |  | Patient scheduling service |
|  | Oncology and Radiotherapy Clinic |  | Department of Legal, Human Resources and General Affairs |
|  | Pulmonary Disease Clinic |  | Department of nutrition and dietetics |
|  | Clinic for Psychiatry |  | Technical Services Department |
|  | Internal Clinic |  | Internal audit service |
|  | Obstetrics and Gynecology Clinic |  | Department of Occupational Safety |
|  | Department of Diagnostic and Interventional Radiology |  | IT Department |
|  | Department of Clinical Microbiology |  | The Directorate of Hospital |
|  | Nuclear medicine department |  |  |
|  | Department of Pathology, Forensic Medicine and Cytology |  |  |
|  | Physical Medicine and Rehabilitation with Rheumatology Division |  |  |
|  | Department of Maxillofacial surgery |  |  |
|  | Department of Medical Laboratory Diagnostics |  |  |
|  | Department of Transfusion medicine |  |  |
|  | Department of Urology |  |  |
|  | Cardiac Surgery Department |  |  |

**What is your highest degree?**

|  | Primary school |  | College - doctor of medicine |
| --- | --- | --- | --- |
|  | High school |  | College - doctor of dental medicine |
|  | Bachelor's degree |  | College – pharmacist |
|  | Master's degree |  | College – other, please specify: |
|  |  |  |  |

**The field in which you have the highest degree:**

|  | Medicine |  | Midwifery |
| --- | --- | --- | --- |
|  | Dental medicine |  | Physiotherapy |
|  | Veterinary medicine |  | Radiologic technology |
|  | Pharmacy |  | Medical laboratory technology |
|  | Nursing |  | Non-medical profession |

**"Do you have a science degree?**

|  | No |
| --- | --- |
|  | Master of Science |
|  | Doctor of Philosophy |

**Working experience?**

|  | Less than a Year |
| --- | --- |
|  | 1-4 years |
|  | 5-10 years |
|  | More than 10 years |

**Do you work with patients daily?**

|  | Yes |
| --- | --- |
|  | No |

**Are you involved in intensive care work daily (for example, ICU, coronary care unit)?**

|  | Yes |
| --- | --- |
|  | No |

**On which location of the University Hospital of Split do you primarily work?**

|  | Firule |
| --- | --- |
|  | Krizine |
|  | Both locations |
|  | Somewhere else, please specify: |

Please enter the **date of response to the questionnaire** (DD/MM/YYYY): _______________________.

**QUESTIONNAIRE – page 1**

**We would like to ask you some questions about the ethical climate in your institution. Please respond indicating how you assess the current situation in your institution, i.e., how strongly you agree with the statements provided. It is important to answer about the current situation, not how you would like it to be. We kindly ask for an honest response.**

"Please mark the answer that best represents your agreement with the statements below."

| **Completely false** | **Mostly false** | **Somewhat false** | **Somewhat true** | **Mostly true** | **Completely true** |
| --- | --- | --- | --- | --- | --- |
| 0 | 1 | 2 | 3 | 4 | 5 |

|  |  | **Completely false** | **Mostly false** | **Somewhat false** | **Somewhat true** | **Mostly true** | **Completely true** |
| --- | --- | --- | --- | --- | --- | --- | --- |
|  | In this company, people are mostly out for themselves. | 0 | 1 | 2 | 3 | 4 | 5 |
|  | The major responsibility for people in this company is to consider efficiency first. | 0 | 1 | 2 | 3 | 4 | 5 |
|  | In this company, people are expected to follow theft own personal and  moral beliefs. | 0 | 1 | 2 | 3 | 4 | 5 |
|  | People are expected to do anything to further the company’s interests. | 0 | 1 | 2 | 3 | 4 | 5 |
|  | In this company, people look out for each other’s good. | 0 | 1 | 2 | 3 | 4 | 5 |
|  | There is no room for one’s own personal morals or ethics in this company. | 0 | 1 | 2 | 3 | 4 | 5 |
|  | It is very important to follow strictly the company’s rules and procedures  here. | 0 | 1 | 2 | 3 | 4 | 5 |
|  | Work is considered sub-standard only when it hurts the company’s interests. | 0 | 1 | 2 | 3 | 4 | 5 |
|  | Each person in this company decides for himself what is right and wrong. | 0 | 1 | 2 | 3 | 4 | 5 |
|  | In this company, people protect their own interest above other considerations | 0 | 1 | 2 | 3 | 4 | 5 |
|  | The most important consideration in this company is each person’s sense  of right and wrong. | 0 | 1 | 2 | 3 | 4 | 5 |
|  | The most important concern is the good of all the people in the company. | 0 | 1 | 2 | 3 | 4 | 5 |
|  | The first consideration is whether a decision violates any law. | 0 | 1 | 2 | 3 | 4 | 5 |
|  | People are expected to comply with the law and professional standards  over and above other considerations. | 0 | 1 | 2 | 3 | 4 | 5 |
|  | Everyone is expected to stick by company rules and procedures. | 0 | 1 | 2 | 3 | 4 | 5 |
|  | In this company, our major concern is always what is best for the other  person. | 0 | 1 | 2 | 3 | 4 | 5 |

*The questionnaire continues on the next page!*

**QUESTIONNAIRE – page 2**

|  |  | **Completely false** | **Mostly false** | **Somewhat false** | **Somewhat true** | **Mostly true** | **Completely true** |
| --- | --- | --- | --- | --- | --- | --- | --- |
|  | People are concerned with the company’s interests—to the exclusion of | 0 | 1 | 2 | 3 | 4 | 5 |
|  | Successful people in this company go by the book | 0 | 1 | 2 | 3 | 4 | 5 |
|  | The most efficient way is always the right way, in this company | 0 | 1 | 2 | 3 | 4 | 5 |
|  | In this company, people are expected to strictly follow legal or professional  Standards. | 0 | 1 | 2 | 3 | 4 | 5 |
|  | Our major consideration is what is best for everyone in the company. | 0 | 1 | 2 | 3 | 4 | 5 |
|  | In this company, people are guided by theft own personal ethics. | 0 | 1 | 2 | 3 | 4 | 5 |
|  | Successful people in this company strictly obey the company policies. | 0 | 1 | 2 | 3 | 4 | 5 |
|  | In this company, the law or ethical code of theft profession is the major  consideration. | 0 | 1 | 2 | 3 | 4 | 5 |
|  | In this company, each person is expected, above all, to work efficiently. | 0 | 1 | 2 | 3 | 4 | 5 |
|  | It is expected that you will always do what is right for the customer and  public. | 0 | 1 | 2 | 3 | 4 | 5 |
|  | People in this company view team spirit as important. | 0 | 1 | 2 | 3 | 4 | 5 |
|  | People in this company have a strong sense of responsibility to the outside  community. | 0 | 1 | 2 | 3 | 4 | 5 |
|  | Decisions here axe primarily viewed in terms of contribution to profit. | 0 | 1 | 2 | 3 | 4 | 5 |
|  | People in this company are actively concerned about the customer’s, and the public’s, interest. | 0 | 1 | 2 | 3 | 4 | 5 |
|  | People are very concerned about what is generally best for employees in the company. | 0 | 1 | 2 | 3 | 4 | 5 |
|  | What is best for each individual is a primary concern in this organization. | 0 | 1 | 2 | 3 | 4 | 5 |
|  | People in this company are very concerned about what is best for themselves. | 0 | 1 | 2 | 3 | 4 | 5 |
|  | The effect of decisions on the customer and the public axe  a primary concern in this company. | 0 | 1 | 2 | 3 | 4 | 5 |
|  | It is expected that each individual is cared for when  making decisions here. | 0 | 1 | 2 | 3 | 4 | 5 |
|  | Efficient solutions to problems are always sought here. | 0 | 1 | 2 | 3 | 4 | 5 |

*Thank you for completing the questionnaire.*


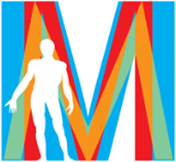

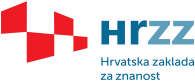


**Sveučilište u Splitu**

**Medicinski fakultet**

**Universitas Studiorum**

**Spalatensis**

**Facultas Medica**

Poštovani,

Cilj ovog upitnika jest utvrditi promjene u procjeni etičke klime zaposlenika KBC Split. Studija se nastavlja na slično istraživanje koje smo proveli na Medicinskom i Filozofskom fakultetu Sveučilišta u Splitu (Viđak M et al, Sci Eng Ethics 2020). Istraživanje je dobilo etička odobrenja odgovarajućih ustanova.

Upitnik ima dva dijela. U prvom dijelu ćemo Vas zamoliti da ispunite nekoliko informacija o sebi, a nakon toga slijedi upitnik koji se odnosi na Vaše stavove o etičkoj klimi na Vašoj ustanovi. Svi podatci iz ovog upitnika prikupljaju se u potpunosti anonimno i bit će analizirani isključivo na grupnoj razini, tako da Vaša anonimnost u nijednom trenutku neće biti ugrožena. Elektronička inačica upitnika neće prikupljati informaciju o Vašoj IP-adresi, što omogućuje potpunu anonimnost.

Daljim ispunjavanjem upitnika dajete svoj pristanak za sudjelovanje u istraživanju.

**Spol** (zaokružite) M Ž **Dob** (u godinama) ______

**Na kojoj sastavnici KBC Split radite?**

|  | Klinika za anesteziologiju, reanimatologiju i intenzivno liječenje |  | Odjel za bolničke infekcije i kliničku epidemiologiju |
| --- | --- | --- | --- |
|  | Klinika za bolesti srca i krvnih žila |  | Odjel za medicinsku fiziku |
|  | Klinika za bolesti uha, nosa i grla s kirurgijom glave i vrata |  | Odjel za osiguranje i unapređenje kvalitete zdravstvene zaštite |
|  | Klinika za dječje bolesti |  | Odjel za znanstveni rad |
|  | Klinika za dječju kirurgiju |  | Služba za centralnu sterilizaciju |
|  | Klinika za infektologiju |  | Služba za čišćenje bolničkog prostora |
|  | Klinika za kirurgiju0 |  | Služba za ekonomsko-financijske poslove |
|  | Klinika za kožne i spolne bolesti |  | Služba za investicije i razvoj (EU projekti) |
|  | Klinika za neurologiju |  | Služba za nabavu |
|  | Klinika za očne bolesti |  | Služba za naručivanje bolesnika |
|  | Klinika za onkologiju i radioterapiju |  | Služba za opće, pravne i kadrovske poslove |
|  | Klinika za plućne bolesti |  | Služba za prehranu i dijetetiku |
|  | Klinika za psihijatriju |  | Služba za tehničke poslove |
|  | Klinika za unutarnje bolesti |  | Služba za unutarnju reviziju |
|  | Klinika za ženske bolesti i porode |  | Odsjek zaštite na radu |
|  | Klinički zavod za dijagnostičku i intervencijsku radiologiju |  | Služba za informatiku |
|  | Klinički zavod za mikrobiologiju i parazitologiju |  | Ravnateljstvo |
|  | Klinički zavod za nuklearnu medicinu |  |  |
|  | Klinički zavod za patologiju, sudsku medicinu i citologiju |  |  |
|  | Zavod za fizikalnu medicinu i rehabilitaciju s reumatologijom |  |  |
|  | Zavod za maksilofacijalnu kirurgiju |  |  |
|  | Zavod za medicinsko laboratorijsku dijagnostiku |  |  |
|  | Zavod za transfuzijsku medicinu |  |  |
|  | Zavod za urologiju |  |  |
|  | Zavod za kardiokirurgiju |  |  |

**Koji je vaš najviši stupanj obrazovanja?**

|  | Osnovna škola |  | Fakultet – doktor medicine |
| --- | --- | --- | --- |
|  | Srednja škola |  | Fakultet – doktor dentalne medicine |
|  | Fakultet – prvostupnička diploma |  | Fakultet – farmacija |
|  | Fakultet – magistar struke |  | Fakultet – ostalo, upišite: |
|  |  |  |  |

**Područje iz kojeg imate najviši stupanj obrazovanja:**

|  | Medicina |  | Primaljstvo |
| --- | --- | --- | --- |
|  | Dentalna medicina |  | Fizioterapija |
|  | Veterinarska medicina |  | Radiološka tehnologija |
|  | Farmacija |  | Medicinska laboratorijska tehnologija |
|  | Sestrinstvo |  | Nezdravstvena profesija |

**Imate li znanstvenu diplomu?**

|  | Ne |
| --- | --- |
|  | Magisterij znanosti |
|  | Doktorat znanosti |

**Koliko dugo radite u KBC Split?**

|  | Manje od 1 godine |
| --- | --- |
|  | 1-4 godine |
|  | 5-10 godina |
|  | Više od 10 godina |

**Radite li svakodnevno s pacijentima?**

|  | Da |
| --- | --- |
|  | Ne |

**Jeste li svakodnevno uključeni u rad intenzivne skrbi (primjerice JIL, koronarna jedinica)?**

|  | Da |
| --- | --- |
|  | Ne |

**Na kojem lokalitetu KBC-a Split pretežno radite?**

|  | Firule |
| --- | --- |
|  | Križine |
|  | Na oba lokaliteta |
|  | Negdje drugdje, navedite: |

Molimo upišite **datum odgovora na upitnik** (DD/MM/GGGG): _______________________.

**UPITNIK – stranica 1**

**Željeli bismo Vas pitati neka pitanja o etičkoj klimi u Vašoj ustanovi.**

**Molimo Vas da odgovorite kako procjenjujete trenutno stanje u Vašoj ustanovi, tj. koliko se slažete s navedenim tvrdnjama. Važno je da odgovorite o tome kakvo je trenutno stanje, a ne kakvo biste željeli da bude. Molimo Vas za iskren odgovor.**

Molimo označite onaj odgovor koji najbolje predstavlja Vaše slaganje s dolje navedenim tvrdnjama.

| **Potpuno netočno** | **Većinom netočno** | **Donekle netočno** | **Donekle točno** | **Većinom točno** | **Potpuno točno** |
| --- | --- | --- | --- | --- | --- |
| 0 | 1 | 2 | 3 | 4 | 5 |

|  |  | **Potpuno netočno** | **Većinom netočno** | **Donekle netočno** | **Donekle točno** | **Većinom točno** | **Potpuno točno** |
| --- | --- | --- | --- | --- | --- | --- | --- |
|  | U ovoj se ustanovi ljudi većinom brinu samo o sebi. | 0 | 1 | 2 | 3 | 4 | 5 |
|  | Glavna odgovornost za ljude u ovoj ustanovi je brinuti se prije svega za učinkovitost. | 0 | 1 | 2 | 3 | 4 | 5 |
|  | U ovoj se ustanovi od ljudi očekuje da se vode svojim osobnim i moralnim uvjerenjima. | 0 | 1 | 2 | 3 | 4 | 5 |
|  | U ovoj ustanovi se od ljudi očekuje da naprave sve kako bi promicali interese ustanove. | 0 | 1 | 2 | 3 | 4 | 5 |
|  | U ovoj ustanovi ljudi paze na dobro drugih ljudi. | 0 | 1 | 2 | 3 | 4 | 5 |
|  | U ovoj ustanovi nema mjesta za osobni moral ili etiku. | 0 | 1 | 2 | 3 | 4 | 5 |
|  | Ovdje je jako važno strogo se voditi pravilima i postupcima ustanove. | 0 | 1 | 2 | 3 | 4 | 5 |
|  | Smatra se da rad nije na razini očekivanog standarda samo kad šteti interesima ustanove. | 0 | 1 | 2 | 3 | 4 | 5 |
|  | Svaka osoba u ovoj ustanovi sama za sebe odlučuje što je ispravno ili krivo. | 0 | 1 | 2 | 3 | 4 | 5 |
|  | U ovoj ustanovi ljudi štite svoje interese više od svega ostalog. | 0 | 1 | 2 | 3 | 4 | 5 |
|  | Najvažniji obzir u ovoj ustanovi je smisao svake osobe za pravdu i nepravdu. | 0 | 1 | 2 | 3 | 4 | 5 |
|  | Najveća briga u ovoj ustanovi je dobro svih ljudi u njoj. | 0 | 1 | 2 | 3 | 4 | 5 |
|  | Prvo što se uzima u obzir u ovoj ustanovi jest je li odluka protivna nekom zakonu. | 0 | 1 | 2 | 3 | 4 | 5 |
|  | Od ljudi se očekuje da se pridržavaju zakona i profesionalnih standarda prije svih drugih obzira. | 0 | 1 | 2 | 3 | 4 | 5 |
|  | Od svakoga se očekuje da se pridržava pravila i postupaka ustanove. | 0 | 1 | 2 | 3 | 4 | 5 |
|  | U ovoj je ustanovi naša glavna briga ono što je najbolje za drugu osobu. | 0 | 1 | 2 | 3 | 4 | 5 |

*Upitnik se nastavlja na sljedećoj stranici!*

**UPITNIK – stranica 2**

|  |  | **Potpuno netočno** | **Većinom netočno** | **Donekle netočno** | **Donekle točno** | **Većinom točno** | **Potpuno točno** |
| --- | --- | --- | --- | --- | --- | --- | --- |
|  | Ljudi se brinu za interese ustanove – do isključenja svega drugoga. | 0 | 1 | 2 | 3 | 4 | 5 |
|  | Uspješni ljudi u ovoj ustanovi pridržavaju se pravila ustanove. | 0 | 1 | 2 | 3 | 4 | 5 |
|  | U ovoj je ustanovi pravi put uvijek onaj koji je najučinkovitiji. | 0 | 1 | 2 | 3 | 4 | 5 |
|  | U ovoj se ustanovi od ljudi očekuje da se strogo pridržavaju pravnih ili profesionalnih standarda. | 0 | 1 | 2 | 3 | 4 | 5 |
|  | Naša je glavna briga ono što je najbolje za svakoga u ustanovi. | 0 | 1 | 2 | 3 | 4 | 5 |
|  | U ovoj se ustanovi ljudi vode svojom osobnom etikom. | 0 | 1 | 2 | 3 | 4 | 5 |
|  | Uspješni ljudi u ovoj ustanovi strogo se pridržavaju pravila ustanove. | 0 | 1 | 2 | 3 | 4 | 5 |
|  | U ovoj su ustanovi glavni obzir zakon ili etički profesionalni kodeks. | 0 | 1 | 2 | 3 | 4 | 5 |
|  | U ovoj se ustanovi očekuje od svake osobe da, iznad svega, radi učinkovito. | 0 | 1 | 2 | 3 | 4 | 5 |
|  | Očekuje se da ćete uvijek napraviti ono što je pravedno za javnost i primatelje Vaših usluga. | 0 | 1 | 2 | 3 | 4 | 5 |
|  | Ljudi u ovoj ustanovi smatraju da je timski duh važan. | 0 | 1 | 2 | 3 | 4 | 5 |
|  | Ljudi u ovoj ustanovi imaju jak osjećaj odgovornosti prema zajednici koja ih okružuje. | 0 | 1 | 2 | 3 | 4 | 5 |
|  | Odluke se ovdje prije svega sagledavaju prema ostvarenju profita. | 0 | 1 | 2 | 3 | 4 | 5 |
|  | Ljudi se u ovoj ustanovi aktivno brinu o interesu primatelja njihovih usluga i interesu javnosti. | 0 | 1 | 2 | 3 | 4 | 5 |
|  | Ljudi se jako brinu o tome što je općenito najbolje za zaposlenike u ustanovi. | 0 | 1 | 2 | 3 | 4 | 5 |
|  | Primarna briga u ovoj ustanovi je ono što je najbolje za svakog pojedinca. | 0 | 1 | 2 | 3 | 4 | 5 |
|  | Ljudi u ovoj ustanovi se jako brinu o onome što je najbolje za njih. | 0 | 1 | 2 | 3 | 4 | 5 |
|  | Učinak odluka na primatelje usluga ove ustanove i na javnost je primarna briga u ovoj ustanovi. | 0 | 1 | 2 | 3 | 4 | 5 |
|  | Ovdje se briga za svakog pojedinca očekuje prilikom donošenja odluka. | 0 | 1 | 2 | 3 | 4 | 5 |
|  | Ovdje se uvijek traže učinkovita rješenja problema. | 0 | 1 | 2 | 3 | 4 | 5 |

*Hvala Vam na ispunjavanju upitnika.*
