# Supplementary material for: Perception of the ethical climate among hospital employees in a public healthcare system: a cross-sectional survey at the University Hospital of Split, Croatia
Source: BMC Med Ethics. 2025 May 7;26:59. doi: 10.1186/s12910-025-01217-1 (PMC12060318; doi:10.1186/s12910-025-01217-1)
Supplement: Supplementary file 3 — Supplementary Material 3: Additional file 3.docx Linear regression analysis of sociodemographic variables [file 12910_2025_1217_MOESM3_ESM.docx]

**Table S1.** Linear regression analysis of sociodemographic variables (enter method)*

| **Predictor by climate** | **Estimate (95% CI)** | **SE** | **t** | ***P*-value** | **R²** | **AIC** | **BIC** |
| --- | --- | --- | --- | --- | --- | --- | --- |
| **Self-interest** |  |  |  |  | 0.0281 | 2194 | 2218 |
| Intercept | 11.5948 (9.772, 13.4179) | 0.9273 | 12.5035 | < .001 |  |  |  |
| Age in years | −0.0676 (−0.114, −0.0216) | 0.0234 | −2.8871 | 0.004 |  |  |  |
| Highest degree | 0.1279 (−0.546, 0.8017) | 0.3427 | 0.3731 | 0.709 |  |  |  |
| Gender | −0.0282 (−0.92, 0.8634) | 0.4535 | −0.0622 | 0.95 |  |  |  |
| Years working | 1.0289 (0.413, 1.6448) | 0.3133 | 3.284 | 0.001 |  |  |  |
| **Friendship** |  |  |  |  | 0.0272 | 2301 | 2325 |
| Intercept | 13.9893 (11.9077, 16.071) | 1.0588 | 13.212 | < .001 |  |  |  |
| Age in years | −0.018 (−0.0706, 0.0345) | 0.0267 | −0.676 | 0.5 |  |  |  |
| Highest degree | −0.2728 (−1.0422, 0.4965) | 0.3913 | −0.697 | 0.486 |  |  |  |
| Gender | −0.2188 (−1.2369, 0.7992) | 0.5178 | −0.423 | 0.673 |  |  |  |
| Years working | −0.533 (−1.2363, 0.1703) | 0.3577 | −1.49 | 0.137 |  |  |  |
| **Personal morality** |  |  |  |  | 0.0162 | 2102 | 2126 |
| Intercept | 9.3649 (7.7398, 10.9899) | 0.8266 | 11.33 | < .001 |  |  |  |
| Age in years | 0.019 (−0.022, 0.0601) | 0.0209 | 0.913 | 0.362 |  |  |  |
| Highest degree | 0.2406 (−0.36, 0.8412) | 0.3055 | 0.788 | 0.431 |  |  |  |
| Gender | 0.1147 (−0.68, 0.9094) | 0.4042 | 0.284 | 0.777 |  |  |  |
| Years working | 0.1876 (−0.3614, 0.7367) | 0.2793 | 0.672 | 0.502 |  |  |  |
| **Company profit** |  |  |  |  | 0.0256 | 2066 | 2090 |
| Intercept | 12.5412 (10.9876, 14.09476) | 0.7902 | 15.87 | < .001 |  |  |  |
| Age in years | −0.0403 (−0.0795, −0.00111) | 0.0199 | −2.021 | 0.044 |  |  |  |
| Highest degree | −0.2835 (−0.8576, 0.2907) | 0.2921 | −0.971 | 0.332 |  |  |  |
| Gender | −0.6417 (−1.4015, 0.11804) | 0.3865 | −1.661 | 0.098 |  |  |  |
| Years working | 0.5542 (0.0293, 1.07902) | 0.267 | 2.076 | 0.039 |  |  |  |
| **Team interest** |  |  |  |  | 0.0307 | 2349 | 2373 |
| Intercept | 12.5741 (10.36289, 14.785) | 1.1247 | 11.18 | < .001 |  |  |  |
| Age in years | 0.0531 (−0.00271, 0.109) | 0.0284 | 1.87 | 0.062 |  |  |  |
| Highest degree | −0.4925 (−1.3097, 0.325) | 0.4157 | −1.18 | 0.237 |  |  |  |
| Gender | −0.7053 (−1.78669, 0.376) | 0.55 | −1.28 | 0.2 |  |  |  |
| Years working | −1.0494 (−1.79647, −0.302) | 0.38 | −2.76 | 0.006 |  |  |  |
| **Company rules** |  |  |  |  | 0.0231 | 2213 | 2237 |
| Intercept | 12.8785 (11.0115, 14.746) | 0.9497 | 13.561 | < .001 |  |  |  |
| Age in years | 0.0601 (0.013, 0.107) | 0.024 | 2.508 | 0.013 |  |  |  |
| Highest degree | −0.3099 (−1, 0.38) | 0.351 | −0.883 | 0.378 |  |  |  |
| Gender | −0.4171 (−1.3302, 0.496) | 0.4644 | −0.898 | 0.37 |  |  |  |
| Years working | −0.8041 (−1.4349, −0.173) | 0.3208 | −2.506 | 0.013 |  |  |  |
| **Efficiency** |  |  |  |  | 0.0267 | 2172 | 2196 |
| Intercept | 13.7637 (11.9907, 15.5368) | 0.9019 | 15.261 | < .001 |  |  |  |
| Age in years | 0.016 (−0.0287, 0.0608) | 0.0228 | 0.704 | 0.482 |  |  |  |
| Highest degree | −0.0708 (−0.7261, 0.5845) | 0.3333 | −0.212 | 0.832 |  |  |  |
| Gender | −1.212 (−2.0791, −0.345) | 0.4411 | −2.748 | 0.006 |  |  |  |
| Years working | −0.4069 (−1.006, 0.1921) | 0.3047 | −1.336 | 0.182 |  |  |  |
| **Social responsibility** |  |  |  |  | 0.0376 | 2211 | 2235 |
| Intercept | 13.9198 (12.0579, 15.7817) | 0.9471 | 14.7 | < .001 |  |  |  |
| Age in years | 0.0579 (0.0109, 0.1049) | 0.0239 | 2.42 | 0.016 |  |  |  |
| Highest degree | −0.5502 (−1.2383, 0.138) | 0.35 | −1.57 | 0.117 |  |  |  |
| Gender | −0.8862 (−1.7968, 0.0243) | 0.4632 | −1.91 | 0.056 |  |  |  |
| Years working | −0.7877 (−1.4167, −0.1586) | 0.32 | −2.46 | 0.014 |  |  |  |
| **Laws and professional codes** |  |  |  |  | 0.0307 | 2165 | 2189 |
| Intercept | 13.5896 (11.8314, 15.348) | 0.8943 | 15.2 | < .001 |  |  |  |
| Age in years | 0.0606 (0.0162, 0.105) | 0.0226 | 2.69 | 0.008 |  |  |  |
| Highest degree | −0.4219 (−1.0717, 0.228) | 0.3305 | −1.28 | 0.202 |  |  |  |
| Gender | −0.4655 (−1.3253, 0.394) | 0.4374 | −1.06 | 0.288 |  |  |  |
| Years working | −0.8484 (−1.4424, −0.254) | 0.3021 | −2.81 | 0.005 |  |  |  |

*Highest degree dummy coded as 1 – High school or lower, 2 – Bachelor's, master's or other university-level degree, 3 – PhD or equivalent. Gender dummy coded as 1 – female, 2 – male. Years working dummy coded as 1 – ≤4 years; 2 – 5–10 years; 3 – >10 years.
